# From Bench to Bedside: Advancements in Precision Oncology and Drug Discovery for Osteosarcoma

**DOI:** 10.3390/cancers18040561

**Published:** 2026-02-09

**Authors:** Luca Giacchi, Elisa Pucci, Nadia Rucci

**Affiliations:** Department of Biotechnological and Applied Clinical Sciences, University of L’Aquila, 67100 L’Aquila, Italy; luca.giacchi@graduate.univaq.it (L.G.); elisa.pucci@graduate.univaq.it (E.P.)

**Keywords:** osteosarcoma, chemotherapy, nanotechnology, immunotherapy, chemoresistance, lung metastases

## Abstract

Osteosarcoma remains a highly aggressive malignancy with limited therapeutic progress and poor outcomes, particularly in metastatic or recurrent cases. Conventional treatment approaches, primarily based on surgery and high-dose chemotherapy, are hindered by significant drawbacks, including severe toxicity, high relapse rates, and drug resistance, underscoring the inadequacy of current standard approaches. This review examines emerging advances in precision medicine and drug discovery, including targeted inhibitors, immunomodulatory agents, combination treatments, and advanced biomaterials, that promise to transform osteosarcoma care. Recent advances, such as combinations of immune checkpoint inhibitors with novel agents or nanoparticle-based drug delivery systems, as well as *CRISPR-Cas9* gene-editing applications, offer new strategies to overcome the inherent challenges of conventional therapies. In addition, cutting-edge research leveraging multi-omics analyses and digital pathology is refining our understanding of the tumour microenvironment, paving the way for more individualised treatment strategies.

## Simple Summary

Osteosarcoma is the most frequent bone tumour in children and young adults. The current strategy for osteosarcoma therapy includes high doses of methotrexate (HD-MTX), doxorubicin, and cisplatin combined with surgery. Established in 1970s, this approach presents several limitations, particularly in metastatic and recurrent cases. This review aims to investigate and highlight emerging discoveries to improve osteosarcoma care. We intend to focus on immunotherapy and target therapy, as well as on nanotechnology-based therapeutic strategies, highlighting the improvements on delivery and distribution of therapy. Moreover, we will shed light on multi-omics approaches which could help in the discovery of new prognostic markers or genetic targets for precision medicine.

## 1. Introduction

Osteosarcoma (OS) is the most prevalent primary malignant bone tumour of childhood and adolescence. The average incidence is 0.3 per 100,000 per year, and it reaches 0.8–1 per 100,000 per year in adolescence [1]. The disease typically originates in the metaphyseal regions of long bones, particularly the distal femur, proximal tibia, and proximal humerus. This predilection reflects the tight coupling between OS biology and the physiology of bone growth [2], where proliferative stresses and microenvironmental cues may favour malignant transformation.

From a biological point of view, although OS is defined by malignant osteoid production, its precise origin remains controversial. Experimental evidence from genetically modified mouse models suggests that OS can arise from cells along the differentiation spectrum from mesenchymal stem cells (MSCs) to more lineage-committed osteoblast precursor cells, reflecting the hierarchical nature of osteoblast differentiation [3]. Several models proposed MSCs as candidate initiating cells for OS due to their capacity to differentiate into multiple mesenchymal lineages, including osteoblasts, chondrocytes, and adipocytes, implying that transformation events in multipotent progenitors could give rise to malignant osteogenic lesions [4]. However, studies using conditional engineered mice have increasingly indicated that cells committed to the osteoblast lineage, particularly osteoprogenitors or pre-osteoblasts, are more efficient at generating OS upon deletion of key tumour suppressors such as p53 and Rb, compared with fully differentiated osteoblasts or uncommitted MSCs [5]. This body of work supports the view that OS frequently originates in an intermediate stage of osteogenic differentiation, where cells retain proliferative potential but have begun lineage commitment. In recent years, transcriptomic and single-cell profiling studies in human OS have further corroborated this concept by showing that tumour cell expression profiles often resemble those of immature osteoblast-lineage cells rather than undifferentiated MSCs; however, definitive identification of the cell of origin in human disease remains technically challenging [6,7,8].

Despite substantial progress in diagnostic imaging and surgical management, clinical outcomes for OS remained essentially unchanged for more than three decades. The introduction of the Multi-Agent Polychemotherapy (MAP) protocol (e.g., high-dose methotrexate, doxorubicin, and cisplatin) initially improved survival for localised disease. However, five-year survival for non-metastatic OS stabilizes around 60–70%, while survival for patients presenting with metastatic disease remains below 30% [9], likely reflecting the inability of current therapies to eradicate micrometastatic disease and the absence of effective systemic options at relapse. Indeed, the greatest challenge in OS management is represented by early metastatic dissemination. Although only 15–20% of patients display detectable metastases at diagnosis, biological evidence indicates that many more harbour occult micrometastases, particularly in the lungs [10], and such occult micrometastases are often resistant to chemotherapy and contribute to relapses. Indeed, lung metastases are responsible for most OS-related deaths [11].

A major barrier to therapeutic progress lies in the biological complexity of OS, which is one of the most genomically unstable human malignancies, displaying widespread chromosomal aneuploidy, chromothripsis-like rearrangements, and extensive copy-number variations [12]. In the context of paediatric malignancies, this degree of genomic instability is highly unusual. Large-scale genomic profiling studies have demonstrated that, whereas most paediatric cancers are characterised by a relatively low mutational burden and simple genomic landscapes driven by recurrent alterations, OS represents a clear outlier [13,14].

**Figure 1:**
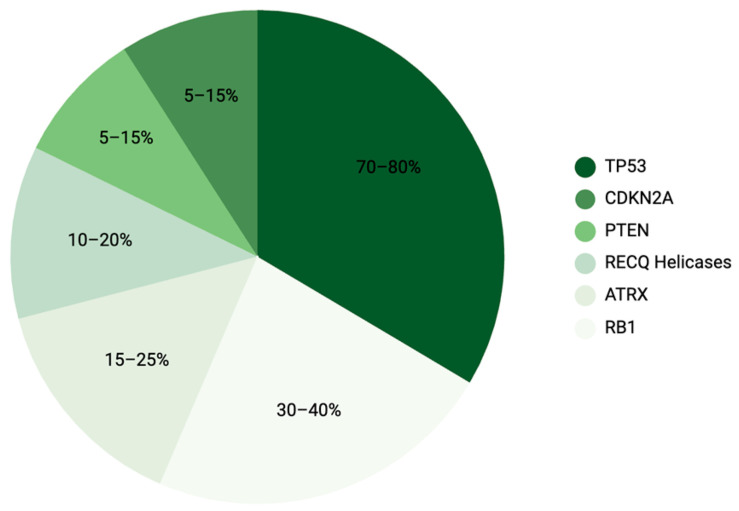
Most frequently altered genes in osteosarcoma. Pie chart illustrates the relative representation of the most commonly altered genes in osteosarcoma based on large-scale genomic sequencing studies. Reported values indicate approximate frequency ranges observed across independent cohorts and are not mutually exclusive, reflecting the extensive genomic instability and co-occurrence of alterations characteristic of osteosarcoma.

Hence, in contrast to tumours driven by recurrent, targetable activating oncogenic mutations, the genetic landscape of OS is characterised by uncontrolled structural alterations and chromosomal instability, with relatively few recurrent activating driver mutations. Instead, OS is characterised by frequent but heterogeneous loss-of-function alterations in tumour-suppressor genes such as *TP53*, *RB1*, *ATRX*, replication-associated DNA helicases, CDKN2, and *PTEN* [15] (Figure 1). These alterations promote aberrant proliferation, impaired DNA-damage responses, and genomic destabilisation, resulting in marked molecular heterogeneity that translates into variable clinical behaviour and substantial interpatient differences in therapeutic sensitivity.

Several oncogenic signalling pathways are recurrently deregulated in OS, including PI3K/AKT/mTOR, Wnt/β-catenin, FGFR, IGF-1R, and Notch. These pathways collectively regulate cellular proliferation, migration, metabolic rewiring, angiogenesis, and resistance to apoptotic and chemotherapeutic stress. Their coexistence and functional redundancy contribute to treatment resistance and complicate the development of targeted therapies. Efforts to inhibit single molecules or pathways have generally failed in clinical trials, as OS cells rapidly activate compensatory signalling networks that sustain survival [16].

**Figure 2:**
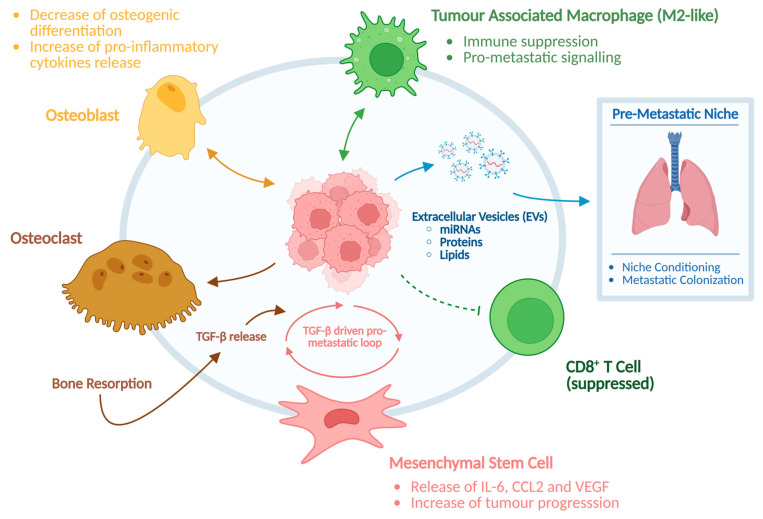
Osteosarcoma tumour microenvironment (TME). Tumour cells engage in reciprocal crosstalk with osteoblasts, leading to impaired osteogenic differentiation and inflammatory reprogramming. Osteoclast-mediated bone resorption results in the release of matrix-stored TGF-β, which activates pro-metastatic signalling pathways in osteosarcoma cells, reinforcing a feed-forward loop that promotes tumour aggressiveness. Mesenchymal stromal cells contribute to tumour progression through the secretion of cytokines and growth factors, including IL-6, CCL2, and VEGF, while tumour-associated macrophages with an M2-like phenotype foster immune suppression and pro-metastatic signalling. Tumour-derived extracellular vesicles transport bioactive molecules, such as miRNAs, proteins, and lipids, reshaping the local microenvironment and contributing to the conditioning of a permissive pre-metastatic niche in the lung. Together, these interconnected processes drive osteosarcoma growth, immune evasion, and early metastatic dissemination. Created in BioRender. Pucci, E. (2026) https://BioRender.com/ke080k7 (accessed on 3 February 2026).

In addition to intrinsic genetic alterations, the tumour microenvironment (TME) plays a pivotal role in OS progression and therapeutic resistance. Bone is a uniquely dynamic environment composed of osteoblasts, osteoclasts, immune cells, mesenchymal stem cells (MSCs), endothelial cells, and bone extracellular matrix components. Interactions between OS cells and the surrounding stroma promote tumour growth, immune evasion, and metastatic dissemination [17]. Osteoclastic bone resorption releases growth factors like transforming growth factor-β (TGF-β), which in turn stimulate tumour proliferation, epithelial mesenchymal transition (EMT)-like conversion, and osteolytic expansion [18]. MSCs within the TME enhance angiogenesis and metastatic competence through the secretion of cytokines such as IL-6, CCL2, and VEGF [19]. Moreover, immune dysregulation is a hallmark of OS: tumour-associated macrophages (TAMs) frequently polarize toward an M2-like phenotype, promoting immunosuppression and metastatic progression (Figure 2) [20].

A growing body of evidence has highlighted a central role for exosomes in mediating communication within the OS microenvironment (Figure 2). Tumour-derived exosomes carry oncogenic proteins, lipids, and RNAs, particularly microRNAs, that remodel stromal compartments, promote osteoclastogenesis, condition the pre-metastatic niche in the lungs, and suppress antitumour immune responses [21]. Parallel advances in the understanding of non-coding RNAs, especially microRNAs and long non-coding RNAs, have revealed complex regulatory networks that modulate gene expression, metastatic behaviour, and chemoresistance [22] (Figure 2).

The convergence of genomic instability, dysregulated signalling, epigenetic reprogramming, and microenvironmental crosstalk explains why OS remains such a challenging malignancy. Indeed, these biological insights have also opened new therapeutic opportunities. Precision oncology approaches leveraging multi-omics analyses, digital pathology, machine learning, and liquid biopsy technologies are beginning to refine patient stratification and enable more rational therapeutic design [23,24]. In parallel, emerging fields such as immunotherapy, targeted molecular inhibitors, nanotechnology-based drug delivery, and biomaterials engineering offer promising avenues to overcome longstanding limitations associated with traditional chemotherapy [25].

### Literature Search Strategy

This narrative review is based on a comprehensive analysis of the available literature. Relevant articles were identified through searches on PubMed, Web of Science, and Google Scholar, using combinations of keywords related to osteosarcoma, precision oncology, targeted therapies, immunotherapy, nanotechnology, and drug resistance. Priority was given to recent and high-quality original research articles and reviews.

## 2. Molecular Pathogenesis and Tumour Microenvironment of Osteosarcoma

Mechanistic studies have shown that OS pathogenesis involves the convergence of several deregulated oncogenic pathways, including PI3K/AKT/mTOR, Wnt/β-catenin, MAPK, FGFR, IGF-1R, and Notch signalling. While Wnt/β-catenin signalling physiologically promotes osteoblast differentiation, its aberrant and sustained activation in OS disrupts the balance between proliferation and terminal osteogenic maturation, thereby maintaining tumour cells in an osteoprogenitor-like state [26]. This altered Wnt output increases proliferation and tumourigenicity rather than normal differentiation. In parallel, hyperactivation of PI3K/AKT enhances apoptosis resistance and contributes to chemoresistance, whereas dysregulated Notch signalling promotes angiogenesis, invasion, and tumour–stroma interactions that reinforce malignant progression [27].

This redundancy among pathways explains why molecularly targeted agents, when used individually, have generally failed to produce significant clinical benefit in OS. Tumour cells readily bypass inhibition of a single node by engaging compensatory signalling networks. Therefore, the molecular architecture of OS strongly supports the need for combination therapies or multimodal strategies that address multiple signalling pathways simultaneously.

### 2.1. The Central Role of TGF-β Signalling in Osteosarcoma Progression

**Figure 3:**
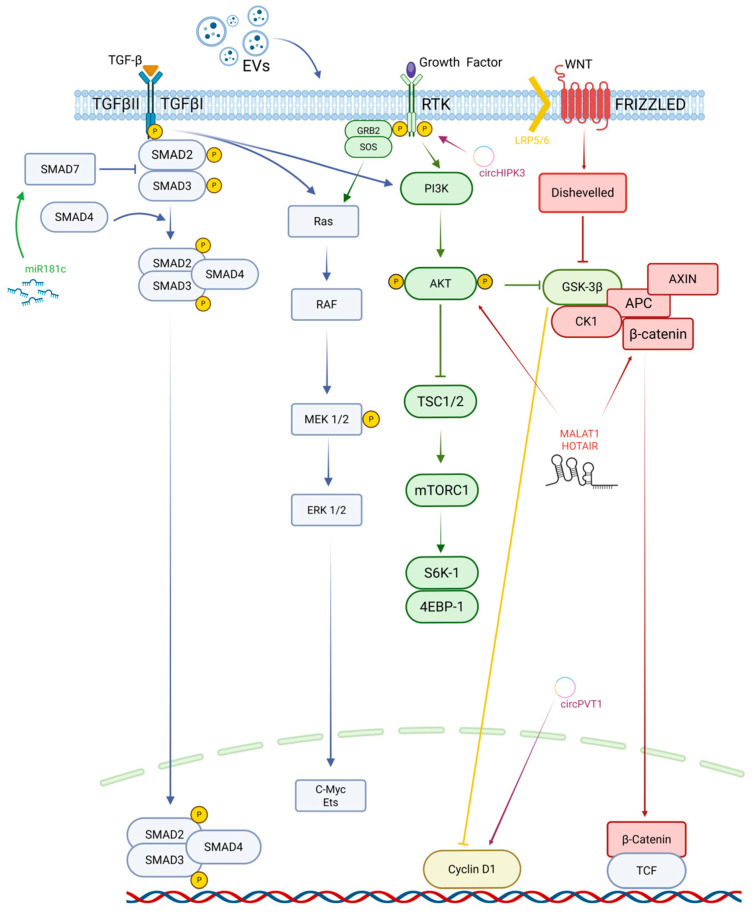
Schematic representation of the interaction among major oncogenic pathways. The figure illustrates the interplay between TGF-β, PI3K/AKT/mTOR, and WNT/β-catenin signalling pathways, as well as the regulatory contribution of extracellular vesicles (EVs) and non-coding RNAs, including circRNAs (purple), lncRNAs (red), and miRNAs (green). Arrows indicate activation, whereas blunt ends indicate inhibition. In canonical TGF-β signalling, ligand binding to TGFβRII leads to recruitment of TGFβRI and phosphorylation of SMAD2/3, which associate with SMAD4 and translocate to the nucleus to regulate gene transcription; SMAD7 negatively modulates this process. In parallel, non-canonical TGF-β signalling activates MAPK and PI3K pathways. Growth factor-activated RTKs stimulate PI3K/AKT signalling, leading to mTORC1 activation and enhanced protein synthesis and cell proliferation, while also promoting cell-cycle progression through GSK3β inhibition. RTK signalling can additionally activate MAPK cascades. In the WNT/β-catenin pathway, WNT ligands bind the Frizzled/LRP5/6 receptor complex, preventing β-catenin degradation and enabling its nuclear translocation to drive transcription of WNT target genes. Created in BioRender. Pucci, E. (2026) https://BioRender.com/qs62xyf (accessed on 3 February 2026).

Among the many deregulated pathways in OS, TGF-β signalling is uniquely significant due to its abundance in the bone microenvironment and its involvement in metastasis. TGF-β is released in substantial quantities during osteoclastic bone resorption, allowing tumour cells to exploit this physiological process to support malignant expansion. Similarly to epithelial tumours where TGF-β may act as a tumour driver, mesenchymal tumours such as OS also respond to TGF-β predominantly through pro-oncogenic programs [28]. TGF-β enhances tumour cell proliferation, activates EMT-like transcriptional signatures, promotes migration and invasion, and regulates angiogenesis. Clinical studies demonstrate that circulating TGF-β levels are significantly increased in OS patients, especially in those with metastatic disease, and correlate with poor prognosis [29]. Mechanistically, TGF-β stimulates the expression of pro-metastatic and osteolytic mediators, including IL-11, MMP-1, CXCR4, CTGF, and PTHrP, all promoting bone degradation, extracellular matrix remodelling and metastatic colonisation of the lungs [30]. These effects are mediated through the canonical SMAD2/3 pathway, as well as non-canonical branches involving JNK, p38 MAPK, and PI3K/AKT, providing multiple layers of signalling redundancy. This biological centrality has prompted significant interest in therapeutically targeting TGF-β [30,31,32] (Figure 3).

### 2.2. Epigenetic Regulation and Non-Coding RNA Networks

Epigenetic regulation has emerged as a major contributor to OS biology, complementing the genomic characteristic of the disease. DNA methylation changes, histone modifications, and especially the activity of non-coding RNAs, such as microRNAs (miRNAs), long non-coding RNAs (lncRNAs), and circular RNAs, shape the transcriptional landscape of OS cells.

MicroRNAs are among the most extensively studied epigenetic regulators in OS. Several tumour-suppressive miRNAs, such as miR-34a and miR-181c, are downregulated in OS, leading to overactivation of oncogenic pathways, including TGF-β signalling. miR-34a promotes differentiation programs and restrains proliferation [33], whereas miR-181c suppresses invasion and metastasis by targeting SMAD7 and attenuating EMT-like transition [34]. miR-23a can inhibit special AT-rich-binding protein 1 (SATB1), thus acting as a tumour suppressor in OS [35]. In vivo experiments conducted by He et al. showed a suppressor role for miR-23a, since its downregulation promotes *RUNX2* and *CXCL12* gene transcription, which in turn foments OS cellular growth, invasion, and migration [36].

In contrast, certain miRNAs act as oncogenic drivers: miR-95-3p, for example, has been associated with aggressive disease and unfavourable prognosis in specific patient cohorts [37]. Another miRNA associated with OS and lung metastasis is miR-106b, which promotes G1/S cell-cycle progression. In vitro experiments on U2OS OS cells showed an alteration of G1/S transition and a reduction of PI3K/AKT and PI3Kp100 pathways expression after miR-106b downregulation [38]. Additionally, miR-95 induces OS growth in vitro and in vivo by targeting sodium channel epithelial 1 Subunit Alph (SCNN1α) [39].

With regard to lncRNAs such as HOTAIR and MALAT1, they have been shown to promote OS invasion and metastatic behaviour by recruiting chromatin-modifying complexes or by acting as competing endogenous RNAs that sequester tumour-suppressive miRNAs [40,41]. Similarly, circHIPK3 and circPVT1 function as miRNA sponges, stabilising oncogenic transcripts and sustaining proliferative and invasive signalling [42,43]. Through these mechanisms, non-coding RNAs contribute to chemoresistance, immune evasion, and metastatic competence. Owing to their pathway-specific and context-dependent activity, non-coding RNAs are increasingly recognised as promising biomarkers and therapeutic targets, with several preclinical studies exploring miRNA mimics, antagomirs, and lncRNA-directed strategies (Figure 3).

### 2.3. Extracellular Vesicle-Mediated Intercellular Communication in Osteosarcoma

Extracellular vesicles (EVs), together with soluble components of the tumour secretome, represent a central mechanism through which OS cells communicate with the bone microenvironment and reshape its biological functions. Rather than acting as isolated carriers of single molecular species, EVs operate as integrated signalling platforms that convey inflammatory, matrix-remodelling, and angiogenic cues, thereby favouring tumour expansion [44].

Experimental studies conducted in our laboratory using EVs released by aggressive OS cells, such as the MNNG/HOS cell line, demonstrated that tumour-derived EVs are efficiently internalised by bone-resident cells and profoundly alter osteoblast function. In osteoblast cultures, exposure to MNNG/HOS-derived EVs suppresses metabolic activity and osteogenic differentiation, as reflected by reduced alkaline phosphatase (Alp) activity and impaired matrix-forming capacity [45]. Concomitantly, OS-EV-treated osteoblasts acquire a pro-inflammatory and tumour-supportive secretory profile, characterised by increased expression and release of cytokines and chemokines involved in bone remodelling and tumour recruitment, including IL-6, IL-1β, and selected CCL and CXCL family members, as well as matrix-remodelling enzymes such as MMP3 [45]. Importantly, these EVs do not directly induce osteoclast differentiation, indicating that their contribution to osteolysis is mediated indirectly through osteoblast-driven inflammatory and paracrine signalling rather than through direct activation of osteoclast precursors.

The biological effects of EVs are reinforced by the broader OS secretome. Prolonged exposure of osteoblasts to conditioned media from MNNG/HOS cells impairs collagen type I deposition and mineralised matrix formation, further weakening bone structural integrity. In addition, tumour-derived soluble factors reshape the protein composition of osteoblast-derived nanoparticles and vesicles, enriching pathways related to extracellular matrix organisation and tissue remodelling. This suggests that OS cells not only deliver instructive signals to bone cells but also reprogram the secretory and vesicular output of the microenvironment to amplify tumour-supportive processes [46].

On the other hand, the impact of OS-derived EVs is not confined to the local tumour microenvironment but extends systemically, favouring lung metastasis through the establishment of a pre-metastatic niche. Evidence from orthotopic, immunocompetent mouse models showed that tumour-derived EVs preferentially accumulate in the lung and are taken up by interstitial macrophages, where they trigger activation of the JAK2/STAT3 pathway and stimulate CXCL2 expression. This signalling cascade promotes the recruitment of granulocytic myeloid-derived suppressor cells, ultimately leading to an increased pulmonary metastatic burden. At the mechanistic level, this pro-metastatic effect was linked to the selective enrichment of S100A11 (a calcium-binding protein of the S100 family involved in inflammatory signalling and tumour progression) within tumour-derived EVs, since genetic depletion of *S100A11* in OS cells or pharmacological blockade of EV release markedly reduced macrophage reprogramming and lung metastasis [47]. Beyond immune modulation, OS-derived EVs also directly influence lung stromal compartments. Both in vitro and in vivo studies demonstrated efficient uptake of tumour EVs by lung fibroblasts, resulting in their activation toward a cancer-associated fibroblast-like phenotype driven by TGF-β1 signalling. Fibroblasts exposed to tumour-derived extracellular vesicles exhibited increased expression of α-SMA, fibronectin, and collagen I, together with enhanced secretion of pro-inflammatory mediators, thereby contributing to extracellular matrix remodelling and the creation of a lung microenvironment permissive to metastatic growth [48] (Figure 3).

## 3. Mechanisms of Metastasis and Chemoresistance in Osteosarcoma

Metastasis and chemoresistance represent the two major obstacles that continue to limit therapeutic success in OS. Although multimodal treatment increases survival for localised disease, most patients who present with metastases or who experience relapse ultimately succumb to progressive lung involvement. The biological processes that drive metastasis and the development of resistance to chemotherapy are multifactorial, involving intrinsic genetic programs, epigenetic alterations, microenvironmental crosstalk, and complex cellular adaptations to stress. Understanding these processes at a mechanistic level is crucial for the development of next-generation therapeutic strategies [49].

### 3.1. Early Dissemination and Lung Tropism

Clinical and molecular evidence suggests that metastatic dissemination in OS often occurs early in the course of disease, even before diagnosis. While only 15–20% of patients present with radiographically detectable metastases, it is widely believed that many harbour micrometastatic lesions at diagnosis [50,51]. These small metastatic deposits, primarily located in the lungs, often evade chemotherapy and eventually give rise to overt metastatic disease. Pulmonary tropism is a defining feature of OS and is driven by both tumour-intrinsic properties and interactions with the lung microenvironment [52].

The dissemination process starts at the primary tumour site, where OS cells acquire motile and invasive phenotypes through EMT-like transitions driven by transcription factors such as Snail, Twist, and ZEB family proteins [53]. Although OS does not undergo classical EMT due to its mesenchymal origin, it activates analogous transcriptional programs that enhance motility, cytoskeletal reorganisation, and extracellular matrix degradation. Upregulation of matrix metalloproteinases (MMP-2 and MMP-9) and cathepsins enables tumour cells to degrade collagen-rich bone matrix and invade surrounding tissues [54,55]. Upon entering the circulation, OS cells face hemodynamic stress and immune surveillance. Their survival is facilitated by resistance to anoikis, enhanced by PI3K/AKT and IGF-1R signalling, and by interactions with platelets, which shield tumour cells and promote extravasation. Upon reaching the lungs, tumour cells exploit a permissive microenvironment shaped by inflammation, vascular permeability, and the presence of tumour-derived exosomes that precondition the lung niche [56].

### 3.2. Molecular Drivers of Metastasis

Several molecular pathways play pivotal roles in promoting metastatic dissemination in OS. Among these, the PI3K/AKT/mTOR axis emerges as a central driver by integrating survival, metabolic adaptation, and motility signals. Activation of PI3K/AKT enhances resistance to oxidative and anoikis-related stress, allowing circulating tumour cells to survive during blood dissemination [57]. In parallel, AKT signalling regulates focal adhesion turnover and integrin-dependent cytoskeletal dynamics, thereby facilitating cell migration, trans-endothelial passage, and extravasation into distant tissues [57]. Importantly, experimental evidence highlights that upstream activation of PI3K leads to enhanced AKT and mTOR phosphorylation, resulting in activation of the mTOR/S6K pathway. This, in turn, promotes increased translation of the EMT-associated transcription factor ZEB1 without altering its mRNA levels, thereby reinforcing migratory plasticity and invasive behaviour. In vivo, genetic disruption of this axis significantly reduces pulmonary metastatic burden, while restoration of ZEB1 expression rescues lung colonisation, demonstrating a causal role for the PI3K/AKT/mTOR–ZEB1 axis in OS lung metastasis [58]. Consistently, aberrant activation of this pathway is frequently observed in OS and reflects both upstream receptor activation and loss of tumour-suppressive control, contributing to metastatic efficiency rather than simply to primary tumour growth.

The Wnt/β-catenin pathway plays a central role in shaping metastatic competence by maintaining stemness-associated transcriptional programs and enabling cellular plasticity [59]. Aberrant activation of β-catenin signalling facilitates the survival of tumour-propagating subpopulations with high self-renewal capacity, increased resistance to stress, and adaptability to foreign microenvironments. This stem-like state is particularly relevant during metastatic colonisation, where OS cells must rapidly adjust to non-bone niches, such as the lung parenchyma [60]. Experimental evidence supports this functional role for Wnt/β-catenin signalling in OS metastatic behaviour: in particular, CCR9-mediated activation of Wnt/β-catenin signalling has been shown to induce cytoskeletal reorganisation and EMT-associated molecular changes, including increased N-cadherin and vimentin expression, which correlate with enhanced migratory and invasive potential in OS models [61]. In parallel, other upstream regulators of Wnt/β-catenin signalling have been implicated in sustaining stem-like properties in OS cells. CD155 has been identified as a modulator of β-catenin activity through activation of SRC/AKT/GSK3β signalling, leading to increased nuclear accumulation of β-catenin and reinforcement of stemness-associated traits, including self-renewal capacity and resistance to cellular stress. This mechanism provides a molecular basis for the maintenance of tumour-initiating subpopulations capable of surviving dissemination and adapting to distant metastatic sites [62].

In parallel, non-coding RNAs represent an additional regulatory layer that fine-tunes metastatic behaviour. As already described, several tumour-suppressive miRNAs, including miR-34a, miR-181c, and miR-143, are frequently downregulated in metastatic OS. Under physiological conditions, these miRNAs restrain invasion and dissemination by targeting genes involved in cytoskeletal remodelling, EMT-like programs, and TGF-β-dependent signalling cascades [63,64,65]. Their loss therefore removes critical brakes on cell motility and invasive capacity. Conversely, a subset of oncogenic miRNAs actively promotes metastatic progression. miRNAs such as miR-1307, miR-501-3p, and miR-20a have been implicated in enhancing OS dissemination through complementary mechanisms that include modulation of extracellular matrix remodelling, activation of osteolytic and inflammatory programs, and adaptation to immune surveillance [66,67,68]. In particular, EV-associated oncogenic miRNAs contribute to the conditioning of a permissive metastatic niche by influencing bone remodelling and stromal responses, thereby linking tumour-intrinsic regulatory circuits to microenvironmental reprogramming [69].

### 3.3. Mechanisms of Chemoresistance

Chemotherapy resistance is a defining feature of OS relapse and metastatic progression and reflects the ability of tumour cells to activate multiple, partially overlapping survival strategies. Osteosarcoma cells display broad resistance mechanisms that involve genetic alterations, epigenetic reprogramming, and dynamic interactions with the tumour microenvironment, which collectively blunt the efficacy of cytotoxic therapies [70]. One of the most established mechanisms of resistance is the overexpression of ATP-binding cassette (ABC) transporters, particularly P-glycoprotein (P-gp, alias ABCB1). Increased ABCB1 expression reduces intracellular accumulation of chemotherapeutic agents such as doxorubicin and paclitaxel, thereby lowering their cytotoxic efficacy. Clinically, elevated ABCB1 levels correlate with poor response to neoadjuvant chemotherapy and reduced overall survival [71].

Alterations in apoptotic signalling further contribute to chemoresistance. Upregulation of pro-survival proteins, including members of the Bcl-2 family and survivin, shifts the balance toward cell survival even in the presence of extensive DNA damage [72]. Concurrently, downregulation of pro-apoptotic factors impairs execution of chemotherapy-induced cell death. These effects are exacerbated by mutations or functional impairment of TP53, which are frequent in OS and compromise DNA-damage sensing, cell-cycle arrest, and apoptotic responses under genotoxic stress [73].

Autophagy represents another key adaptive mechanism that could support survival during chemotherapy exposure. Although autophagy can exert context-dependent tumour-suppressive effects, in OS, it often functions as a cytoprotective response to drug-induced stress. Enhanced autophagic flux enables tumour cells to recycle damaged organelles and macromolecules during treatment with agents such as cisplatin and doxorubicin. Inhibition of autophagy has been shown to restore chemosensitivity in multiple preclinical models, highlighting its role as a compensatory survival pathway [74].

Epigenetic mechanisms further reinforce resistance phenotypes. Changes in DNA methylation patterns and dysregulation of non-coding RNAs reshape transcriptional programs associated with survival and stress tolerance [75]. Loss of tumour-suppressive miRNAs, including miR-34a and miR-181c, leads to sustained activation of pro-survival and DNA repair pathways, while overexpression of oncogenic miRNAs promotes drug efflux, metabolic adaptation, and resistance to apoptosis. These epigenetic alterations provide plasticity, allowing OS cells to rapidly adapt to chemotherapy-induced selective pressure.

The TME plays a crucial amplifying role in chemoresistance. Dense extracellular matrix deposition limits drug penetration, while hypoxic conditions reduce reactive oxygen species (ROS)-mediated cytotoxicity and favour activation of hypoxia-responsive survival programs. Moreover, crosstalk between OS cells and stromal or immune components, such as MSCs and M2-polarised macrophages, activates pro-survival signalling pathways, including STAT3 and NF-κB. These microenvironment-driven signals further protect tumour cells from chemotherapy-induced damage and contribute to the persistence of resistant subclones [76]. All together, these mechanisms highlight that chemoresistance in OS arises from the integration of tumour-intrinsic adaptations and microenvironmental support, rather than from a single dominant alteration.

## 4. Current Standard-of-Care Therapies and Their Limitations

Neoadjuvant chemotherapy or adjuvant chemotherapy combined with surgical resection of primary tumour has been the standard treatment of OS for more than three decades. Established in the 1970s and 1980s, this multimodal approach improved survival in patients with no metastatic disease [77]. Differently, a poor long-term survival rate was associated with metastatic or recurrent OS. Outcomes for these patients remain unchanged despite refinements in surgical techniques and supportive care [78]. Chemotherapeutic regimen, commonly referred to Multi-Agent Polychemotherapy (MAP), includes high-dose methotrexate (HD-MTX), doxorubicin, and cisplatin [79]. The overall survival in paediatric and adolescent patients is improved by high-dose methotrexate. However, the nephrotoxic, hepatotoxic, and mucositis effects resulting from the use of HD-MTX require vigilant monitoring during treatment [80]. Moreover, cisplatin is associated with severe nephrotoxicity, ototoxicity, and neurotoxicity [81], while cumulative dose-dependent cardiotoxicity is caused by doxorubicin [82]. These toxicities impose significant limitations on treatment intensification, particularly in young patients.

Additional agents, such as ifosfamide and etoposide, are frequently used in patients with relapsed or refractory disease. These drugs are commonly administered in combination (ifosfamide–etoposide (IE) regimen) to exploit complementary mechanisms of DNA damage induction and cell-cycle interference [83]. While these regimens can induce temporary disease control or partial responses in a subset of patients, especially in the metastatic or recurrent cases, their overall impact on long-term survival remains limited. However, their clinical use is often accompanied by cumulative toxicity, including myelosuppression, nephrotoxicity, and secondary malignancy risk [84]. Histological evaluation of tumour necrosis following neoadjuvant chemotherapy remains one of the strongest prognostic factors in OS; patients with <90% necrosis (poor responders) have significantly lower survival rates compared to good responders. However, even in good responders, relapses remain common. Intensifying chemotherapy has not consistently improved outcomes, underscoring the complexity of resistance mechanisms and the inadequacy of cytotoxic escalation as a long-term strategy [85].

Looking at surgical resection, it remains essential for local control. Limb-salvage surgery, endoprosthetic reconstruction, and microsurgical techniques have become a substitution for amputation and improve postoperative function [86]. Nevertheless, surgery alone cannot eradicate micrometastatic disease, and local recurrence remains a concern in poor histologic responders or in cases where clear surgical margins cannot be reliably achieved [87]. Furthermore, the complicated resectability of axial or pelvic tumours contributes to poorer outcomes [88]. Radiation therapy (RT) can be useful as adjuvant treatment in specific cases, such as postoperative treatment of close or positive surgical margins [89]. Traditional RT uses high-energy X-rays to cause cells’ death through damage on DNA, but high DNA repairing seems to be present in patients [90]. Although new technologies, such as proton therapy or carbon-ion therapy, improve dose delivery and facilitate precise targeting of OS cells, the impact of RT remains modest in comparison to its success in other sarcomas [91].

Another major limitation of the current standard of care is the largely uniform application of this therapy across biologically heterogeneous tumours. The standard MAP regimen is applied in most of the cases, despite growing evidence that OS encompasses multiple molecular subtypes with distinct biological behaviours and treatment responses. The emergence of precision oncology, supported by genomic, transcriptomic, and epigenomic profiling, could represent a starting point for patients’ stratification and identification of novel therapeutic agents [3].

Disease relapse remains one of the most unsolved problems in OS; in most cases, recurrence develops within the first two years after diagnosis and is associated with a dismal prognosis, with long-term survival rates rarely exceeding 20%. Once relapse occurs, therapeutic options are fewer. Treatment often consists of reusing cytotoxic agents already administered during first-line therapy, empirical use of off-label targeted drugs, or referral to early-phase clinical trials [92]. Surgical removal of metastatic lesions, most commonly through lung metastasectomy, can provide a survival benefit in carefully selected patients, but it is seldom curative when systemic, therapy-resistant disease has already emerged [93].

## 5. Targeted Therapies in Osteosarcoma

Osteosarcoma is marked, as mentioned before, by extensive genomic instability, a relatively low burden of recurrent point mutations, with the lack of a single dominant, druggable driver alteration. However, molecular profiling and functional studies are nowadays increasingly pointing to pathway dependencies that may be exploitable, especially when targeted agents are used in rational combinations rather than as stand-alone treatments.

### 5.1. Targeting Receptor Tyrosine Kinases and Downstream Pathways

Receptor tyrosine kinases (RTKs) such as IGF-1R, PDGFR, VEGFR, FGFR, and MET are frequently deregulated in OS, contributing to tumour proliferation, angiogenesis, and metastatic competence. Several studies were promising on IGF-1R inhibition, given the strong preclinical evidence linking IGF-1R signalling to OS growth and survival [94]. However, multiple clinical trials testing monoclonal antibodies or small-molecule IGF-1R inhibitors failed to demonstrate significant clinical benefit, likely due to pathway redundancy and compensatory signalling through insulin receptor isoforms [95].

VEGFR and PDGFR inhibitors, including pazopanib and regorafenib, have demonstrated modest activity in relapsed OS. Regorafenib, a multikinase inhibitor targeting VEGFR1–3, KIT, RET, and RAF kinases, seems to allow an improvement in progression-free survival in randomised phase II trials and is now considered one of the few targeted agents with reproducible activity in refractory OS. Nevertheless, responses remain transient, and its benefit is primarily cytostatic rather than cytotoxic [96]. Sorafenib is another inhibitor that has been tested in OS for several years, largely because of its activity on multiple kinases that are relevant to bone tumours [97]. In practice, however, its use as a single agent has not been translated into clear or durable clinical benefit. Most of the interest around sorafenib has come from combination studies rather than from monotherapy trials. For instance, when sorafenib was administered together with the mTOR inhibitor everolimus, some heavily pretreated patients experienced longer periods of disease stabilisation [98]. Similar considerations could be extended to its combination with denosumab, which has been reported to produce clinical benefit in a limited number of cases [98].

From a biological standpoint, the PI3K/AKT/mTOR pathway represents an obvious target, given its frequent activation in OS patients. Nevertheless, clinical experience with mTOR inhibitors such as everolimus or temsirolimus has been variable. In some patients, short-term disease control has been observed, while in others, the effect was minimal or absent. This heterogeneity likely reflects the ability of tumour cells to rapidly develop compensatory signalling mechanisms, a phenomenon that has been repeatedly observed in both preclinical models and clinical settings [99]. Therefore, current therapeutic efforts are increasing on combination strategies, including dual PI3K/mTOR inhibition or the addition of receptor tyrosine kinase inhibitors, rather than further evaluation of single-agent mTOR blockade (Table 1).

### 5.2. Targeting TGF-β Signalling

Several pharmacological approaches have been developed to interfere with TGF-β signalling, including neutralising antibodies, ligand traps, antisense oligonucleotides, and small-molecule inhibitors targeting the type I receptor kinase (ALK5) [98]. Among these, the ALK5 inhibitor vactosertib is one of the most investigated agents; indeed, in vivo experiments using murine and xenograft OS models showed that vactosertib treatment reduces primary tumour growth and lung metastatic burden while significantly inhibiting SMAD2 phosphorylation in tumour tissues [100]. Notably, vactosertib administration was also associated with increased infiltration of CD4^+^ and CD8^+^ T cells and natural killer cells, together with a reduction in regulatory T cells and M2-like macrophages within the tumour microenvironment, indicating a shift toward a less immunosuppressive milieu [100].

Looking at clinics, early-phase trials evaluating TGF-β pathway inhibitors across multiple solid tumours and hematologic malignancies suggest that these agents are generally tolerable. For example, vactosertib has demonstrated an acceptable safety profile in phase I/II studies, with the most frequently reported treatment-related adverse events including fatigue, abdominal pain, and transient elevations in liver enzymes, while serious toxicities were infrequent [101]. These safety data have provided the rationale for exploring combination strategies rather than single-agent use. Combining TGF-β inhibition with immune checkpoint blockade has been proposed to counteract TGF-β-mediated immune exclusion; however, early clinical experience in OS remains limited, and objective responses have so far been modest [102] (Table 1).

### 5.3. Targeting Cell-Cycle and DNA Damage-Response Pathways

Osteosarcoma is characterised by extensive genomic instability, a feature that has naturally drawn attention to DNA damage response (DDR) pathways as potential therapeutic targets [103]. Rather than reflecting isolated defects, genomic-profiling studies indicate that, in many cases, OS harbours alterations across multiple DDR components, including *TP53*, *ATM*, *ATR*, and genes involved in homologous recombination and replication stress regulation [104]. As a result, tumour cells often rely on compensatory repair mechanisms instead of complete loss of DDR function. This pattern of widespread but heterogeneous DDR dysfunction distinguishes OS from classical *BRCA*-driven tumours and makes direct application of established synthetic lethal strategies more challenging [105]. PARP inhibitors are the most clinically advanced agents targeting DDR pathways, but their activity in OS remains uncertain. Canonical *BRCA1* or *BRCA2* mutations are uncommon in this disease; however, functional studies suggest that a subset of OS displays impaired homologous recombination and elevated replication stress [106,107]. In preclinical models, PARP inhibition alone generally produces limited cytotoxic effects, whereas a clearer impact is observed when PARP inhibitors are combined with DNA-damaging treatments such as cisplatin or ionising radiation [108].

Disruption of cell-cycle control represents another potential way to exploit DDR defects in OS. Alterations affecting the RB pathway, including RB1 loss or functional impairment of G1/S checkpoint regulation [109], are reported in a considerable fraction of tumours and contribute to unchecked proliferation and replication stress [110]. Based on this rationale, CDK4/6 inhibitors have been tested in OS preclinical models. These studies show that CDK4/6 blockade can induce G1 arrest, slow tumour cell proliferation, and increase sensitivity to DNA-damaging chemotherapy by restricting repair capacity during replication [111]. At the same time, translation to the clinic remains uncertain, as OS shows marked heterogeneity in RB pathway status, and preserved RB function appears necessary for sustained responses to CDK4/6 inhibition [112]. Overall, current data indicate that DDR- and cell–cell-targeted strategies in OS are unlikely to provide meaningful benefit when used as single agents. Their potential appears greater in combination settings, particularly when treatments are designed to exploit replication stress, defective checkpoint control, or chemotherapy-induced DNA damage (Table 1).

### 5.4. Epigenetic Therapies: HDAC and DNMT Inhibitors

Among the different classes of epigenetic agents, histone deacetylase (HDAC) inhibitors have been extensively studied. Broad-spectrum compounds such as vorinostat and panobinostat consistently reduce OS cell viability in vitro, inducing cell-cycle arrest and apoptosis and markedly impairing clonogenic growth [113]. When evaluated as single agents in vivo, however, their antitumour effects are generally limited and often accompanied by systemic toxicity [114]. This has shifted attention toward more selective approaches and combination strategies. In this context, simultaneous inhibition of HDAC4 and HDAC8 using tasquinimod and PCI-34051 has been shown to significantly enhance the activity of doxorubicin in aggressive OS models. In SJSA-1 cells, this combination reduced cell viability by roughly 55–60% and nearly abolished colony formation, while increasing apoptotic markers such as cleaved caspase-3 and caspase-8 [115]. Notably, comparable effects were observed in three-dimensional spheroid systems, where combined treatment decreased spheroid volume by more than 30% and limited migratory behaviour, suggesting an impact that extends beyond simple growth inhibition [115]. At the molecular level, these effects were associated with reduced RUNX2 expression and attenuation of PI3K/AKT signalling, two pathways closely implicated in OS progression and resistance to chemotherapy.

DNA methyltransferase (DNMT) inhibitors have also been investigated in OS, particularly in models of drug resistance. As single agents, demethylating compounds such as decitabine or 5-aza-2′-deoxycytidine generally exert only modest antiproliferative effects. Their activity becomes more evident when they are combined with HDAC inhibitors, leading to a more sustained suppression of tumour cell growth. In chemo-resistant OS cells, this combined epigenetic targeting has been shown to reactivate silenced apoptotic programs and partially restore sensitivity to cytotoxic stress, indicating that DNA methylation and histone acetylation cooperate in maintaining resistant phenotypes [116]. In addition to direct effects on tumour cell survival, DNMT inhibition appears to influence tumour immunogenicity. Interestingly, in OS models, overexpression of DNMT1 has been associated with repression of CXCL12, TAP1, and LMP2, resulting in reduced CD8^+^ T-cell infiltration and immune escape [117,118]. Pharmacological demethylation reverses these changes, restoring chemokine expression and enhancing antitumour immune responses in preclinical systems. Together, these observations provide a mechanistic basis for combining epigenetic agents with immunotherapy or other immune-modulating strategies.

Overall, available experimental data suggest that epigenetic therapies do not deliver durable benefits as monotherapies in OS. Their main contribution could probably be achieved in combination settings, where epigenetic modulation can reshape transcriptional programs, weaken resistance mechanisms, and increase responsiveness to chemotherapy or immunotherapy. Hence, future translational efforts should focus on identifying epigenetic biomarkers and developing combination therapies (Table 1).

### 5.5. CRISPR-Cas9 Editing: Recent Achievements and Therapeutical Strategies

In OS, CRISPR–Cas9 technology has been used primarily as a functional approach rather than as a direct therapeutic tool. Because of the marked genomic instability of this disease and the absence of recurrent, dominant driver mutations, CRISPR-based gene editing has provided a practical way to identify genes that are truly required for tumour cell proliferation, survival, and drug response, going beyond correlative genomic observations. An early application of CRISPR in OS addressed immune-related pathways. Using CRISPR/Cas9-mediated knockout of *PD-L1*, Liao et al. edited the OS cell lines KHOS and MNNG/HOS. *PD-L1* loss did not significantly affect cell migration or invasion, but it resulted in a clear reduction in cell proliferation and clonogenic growth [119]. Importantly, *PD-L1*-deficient cells showed increased sensitivity to standard chemotherapeutic agents. In these models, the IC_50_ for doxorubicin decreased from approximately 0.09 μM in control cells to about 0.03 μM after *PD-L1* deletion, while sensitivity to paclitaxel was similarly enhanced [119]. These findings indicated that PD-L1 contributes to chemoresistance through cell-intrinsic mechanisms, independently of its role in immune checkpoint signalling, and highlighted the value of CRISPR editing in uncovering non-obvious functional dependencies.

More recently, larger-scale CRISPR screening approaches have been applied to systematically map essential genes in OS. In a kinome-wide CRISPR–Cas9 loss-of-function screen performed across multiple OS models, including U2OS, Saos-2, and OS-732 cells, Wang et al. identified Polo-like kinase 1 (*PLK1*) as the top common and essential gene across the OS cell lines. Genetic disruption of *PLK1* has a strong antiproliferative effect, markedly reducing clonogenic survival in vitro and inducing G2/M cell-cycle arrest. This was accompanied by accumulation of DNA damage markers, including γ-H2AX, and activation of caspase-dependent apoptosis [120]. The relevance of *PLK1* was confirmed in vivo. CRISPR-mediated *PLK1* knockout significantly suppressed tumour growth in xenograft and patient-derived xenograft models, with tumour volume and weight reduced by approximately 50–60% compared to control tumours. In addition, *PLK1* loss was associated with a reduction in pulmonary metastatic burden. Importantly, no significant histological toxicity was observed in major organs [120]. These genetic findings were further validated pharmacologically. Treatment with the *PLK1* inhibitor volasertib recapitulated the effects of genetic PLK1 deletion, inducing G2/M arrest, increasing γ-H2AX and CHK2 phosphorylation, and promoting apoptosis [121]. Weekly in vivo administration of volasertib led to a marked reduction in tumour growth, decreased Ki-67 staining, and increased TUNEL positivity, again without overt systemic toxicity [121]. The close correspondence between genetic and pharmacological inhibition reinforces the translational relevance of the CRISPR screen and supports PLK1 as a functionally therapeutic target in OS.

However, despite these advances, direct therapeutic genome editing remains ethically and technically difficult to apply in clinics for OS. Efficient tumour-specific delivery, off-target effects, and long-term safety remain substantial challenges. At present, the most realistic contribution of CRISPR technology relies on defining functional gene dependencies and prioritising druggable targets, rather than in direct genome editing of tumour cells. In this context, CRISPR-based functional screening represents a powerful strategy to bridge the gap between genomic complexity and rational therapeutic development in OS (Table 1).

### 5.6. Translational Limitations and Future Perspectives of Targeted Therapies in Osteosarcoma

Despite extensive preclinical evidence supporting multiple molecular targets in OS, the clinical impact of targeted therapies has remained limited. This gap largely reflects the pronounced biological heterogeneity of the disease, the absence of a dominant oncogenic driver, and the high degree of signalling redundancy that allows tumour cells to rapidly activate compensatory pathways following single-agent inhibition. Consequently, strategies targeting receptor tyrosine kinases, PI3K/AKT/mTOR, TGF-β signalling, DNA damage response pathways, or epigenetic regulators have generally produced modest and transient responses when used as monotherapies.

Beyond biological complexity, translational progress is further limited by regulatory and practical challenges. Safety considerations are particularly relevant in paediatric and adolescent populations, where long-term toxicity remains a major barrier. In addition, manufacturing complexity, costs associated with combination regimens, and the lack of validated predictive biomarkers limit patient selection and trial optimisation. Future advances will likely depend on rational combination approaches, biomarker-driven stratification, and functional target prioritisation strategies, rather than further evaluation of single-pathway inhibitors.

## 6. Immunotherapy and Immune Modulation in Osteosarcoma

Immunotherapy has transformed the treatment of several cancers, but similar progress has not been achieved in OS. Although there is evidence that OS cells can express tumour-associated antigens and that immune cells are present within the tumour tissue, clinical responses to immunotherapeutic approaches have largely failed. In practice, osteosarcoma’s behaviour induces the generation of an “immune-cold tumour”, with limited cytotoxic T-cell infiltration, dominant immunosuppressive signals, and a tumour microenvironment that actively restricts effective immune activation. This mismatch between apparent immunogenic features and poor therapeutic responsiveness highlights how the immune biology of OS is still not fully elucidated.

### 6.1. The Immunological Landscape of Osteosarcoma

The immune microenvironment of OS is characterised by the presence of different immunosuppressive cellular populations: among these, tumour-associated macrophages (TAMs) represent one of the most abundant immune subsets [122]. Immunohistochemical and transcriptomic analyses show a prevalence of macrophages polarised toward an M2-like phenotype, which actively contribute to immune suppression and tumour progression. In clinical cohorts of OS patients, high M2 macrophage infiltration correlates with reduced overall survival and increased metastatic risk, whereas tumours enriched in M1-polarised macrophages display improved antigen presentation capacity and higher expression of T-cell-activating signals [122].

Comprehensive immunogenomic profiling has further clarified the extent of immune dysfunction in OS. In an integrated multi-omics analysis of primary, recurrent, and metastatic OS samples, low immune infiltration scores have been reported when compared with tumours known to respond to immune checkpoint blockade. In this cohort, CD8^+^ T cells accounted for only a minor fraction of infiltrating immune cells, and T-cell receptor (TCR) analysis revealed limited clonality, indicating poor antigen-driven T-cell expansion. Notably, fewer than one-third of predicted neoantigens were transcriptionally expressed. This was mainly explained by increased nonsense-mediated decay, which degraded neo-antigenic transcripts and thereby reduced antigen presentation, even in the presence of extensive structural genomic alterations [123]. In parallel, regulatory T cells were consistently enriched within the OS microenvironment and contributed to immune suppression through sustained IL-2 consumption and secretion of inhibitory cytokines such as TGF-β. This imbalance between effector and regulatory lymphocyte populations was accompanied by impaired dendritic cell and natural killer (NK) cell activity. Functional analyses showed reduced expression of antigen-processing and presentation machinery, including TAP1 and LMP2, as well as increased expression of inhibitory ligands on tumour cells, further limiting immune effectiveness [123].

### 6.2. Immune Checkpoint Inhibitors: Clinical Evidence and Biological Limitations

Although immune checkpoint inhibitors (ICIs) have transformed the management of several solid malignancies, their translation into meaningful clinical benefit for OS patients has been challenging. Clinical trials evaluating PD-1/PD-L1 or CTLA-4 blockade have consistently shown limited efficacy. For instance, in the SARC028 study, pembrolizumab induced clear responses in only a small fraction of OS patients, with most individuals experiencing disease stabilisation or progression rather than tumour regression [124]. Comparable findings have been reported with other agents such as nivolumab, atezolizumab, and ipilimumab, which demonstrated acceptable safety profiles but minimal therapeutic impact when used as monotherapy [125].

The modest clinical activity of ICIs can be largely explained by the unique biological features of OS. This tumour is generally considered immunologically “cold”, characterised by a low tumour mutational burden and, consequently, a limited repertoire of neoantigens capable of eliciting robust T-cell responses. In parallel, defects in antigen processing and presentation are often due to reduced MHC class I expression, further impairing immune recognition [126]. Beyond tumour-intrinsic factors, the OS microenvironment plays a central role in limiting immunotherapy efficacy. It is dominated by immunosuppressive cell populations, including regulatory T cells, myeloid-derived suppressor cells, and tumour-associated macrophages, which collectively dampen cytotoxic T-cell function and promote immune escape. Moreover, PD-L1 expression is highly heterogeneous and frequently low, reducing the possibility of a consistent response to PD-1/PD-L1 blockade [126] (Table 2).

### 6.3. Macrophage-Targeted Immunotherapy

Macrophages constitute a major immune component of the OS microenvironment and exert a profound influence on disease behaviour. Their abundance and functional plasticity have progressively shifted therapeutic interest from strategies that exploit macrophage biology to counteract tumour progression [127,128]. Within this framework, mifamurtide (liposomal muramyl tripeptide phosphatidylethanolamine, L-MTP-PE) represents the most established macrophage-directed intervention in OS. Acting as an immunostimulatory agent derived from bacterial wall structures, mifamurtide activates monocytes and macrophages and induces the production of pro-inflammatory cytokines, including TNF-α and IL-1β [129]. This activation enhances macrophage-mediated antitumoural activity, and when mifamurtide is administered alongside conventional chemotherapy, it has been associated with improved overall survival and a favourable trend in event-free survival among OS patients [129].

Other pharmacological strategies focus on limiting the protumoural activity of TAMs. Interfering with the CSF-1/CSF-1R signalling pathway, which is essential for macrophage recruitment and survival, has emerged as a means to reduce TAM presence within the tumour microenvironment and to improve responses to additional anticancer treatments. At the same time, the inherent adaptability of macrophages has opened the door to reprogramming approaches aimed at redirecting TAMs away from an M2-like immunosuppressive state [128]. Experimental evidence indicates that such phenotypic shifts can restrain OS growth and metastatic spread. In this setting, all-trans retinoic acid (ATRA) has been shown to suppress M2 polarisation and limit metastatic progression, whereas metformin has been reported to modify macrophage polarisation and metabolic programs, ultimately contributing to tumour growth inhibition [130].

A further macrophage-centred therapeutic strategy targets the CD47/SIRPα innate immune checkpoint. CD47 functions as a “don’t eat me” signal that prevents macrophage-mediated phagocytosis and is frequently upregulated in OS cells. Disrupting the interaction between CD47 and SIRPα restores macrophage phagocytic capacity and reduces tumour growth in preclinical OS models. Despite the compelling biological rationale, clinical investigations targeting this pathway in OS patients have not yet been elucidated [131] (Table 2). Overall, these approaches underscore the central role of macrophages in shaping the OS microenvironment and support their relevance as therapeutic targets.

### 6.4. CAR-T and CAR-NK Cell Therapies

Osteosarcoma highlights many of the intrinsic limitations of this approach, since one of the most persistent obstacles is antigen selection: many surface molecules expressed by OS cells are not tumour-exclusive and are also detected, to varying degrees, in normal tissues, which inevitably raises limitations about on-target off-tumour toxicity [127,132]. Even when candidate antigens are identified, therapeutic efficacy is further compromised by tumour heterogeneity, inefficient trafficking of infused cells, early functional exhaustion, and poor long-term persistence within the strongly immunosuppressive OS microenvironment [132].

Within this challenging landscape, several tumour-associated antigens have nonetheless been pursued as CAR-T targets: notably, HER2, GD2, and B7-H3 (CD276). Clinical experience with HER2-directed CAR-T cells in OS and related sarcomas has consistently demonstrated an acceptable safety profile, with no evidence of severe cytokine release syndrome or clinically meaningful off-tumour toxicity. However, antitumour activity has generally been limited, and durable responses have been uncommon, a finding that has been largely attributed to antigen heterogeneity and progressive CAR-T cell dysfunction following tumour infiltration [133]. GD2, a disialoganglioside enriched in paediatric solid tumours, including OS, has provided a strong biological rationale for CAR targeting. While GD2-specific CAR-T cells showed pronounced cytotoxicity in preclinical models, early clinical evaluation revealed only transient tumour regression in a subset of patients, without sustained benefit. These results underscore the relevance of immune escape mechanisms and insufficient in vivo persistence as major barriers to effective clinical translation [134]. In contrast, increasing attention has focused on B7-H3, which is highly expressed in OS while showing limited distribution in normal tissues. Preclinical studies on paediatric solid tumour models (Patient- and Cell Line-Derived Xenograft, PDX and CDX) have reported marked tumour regression in OS models treated with B7-H3-targeted CAR-T cells [135]. The translational relevance of this antigen is further supported by data from B7-H3-directed antibody–drug conjugates, which achieved an overall response rate of 92.3%, with 61.5% of paediatric solid tumour models maintaining complete responses. Early-phase clinical trials investigating B7-H3 CAR-T cells are currently underway, and preliminary observations suggest biological activity; however, long-term efficacy remains to be determined [135].

Beyond CAR-T strategies, CAR-engineered natural killer (CAR-NK) cells are increasingly being explored as an alternative adoptive cell platform for OS. Compared with CAR-T cells, CAR-NK cells are associated with a lower risk of cytokine release syndrome and graft-versus-host disease, and they retain intrinsic cytotoxic activity that is less dependent on antigen presentation through major histocompatibility complex molecules [136]. Preclinical studies have demonstrated that CAR-NK cells targeting OS-associated antigens such as B7-H3, CD70, and MCAM can effectively suppress tumour growth. Nevertheless, the short lifespan and limited in vivo persistence of adoptively transferred NK cells continue to represent significant challenges [136].

Taken together, current evidence suggests that CAR-based cellular therapies hold considerable promise for OS but remain constrained by biological resistance mechanisms intrinsic to solid tumours. Progress in this field will likely depend on improved antigen-selection strategies to enhance immune cell trafficking and persistence, and the rational integration of CAR-T and CAR-NK approaches into combination therapeutic regimens (Table 2).

### 6.5. Overcoming Immune Resistance in Osteosarcoma: Future Perspectives

Overall, these observations indicate that the limited efficacy of immunotherapy in OS is not due to a lack of potential immunogenicity, but rather to dominant immune-evasion mechanisms operating at both tumour-intrinsic and microenvironmental levels. From this perspective, immune checkpoint blockade alone is unlikely to be sufficient. Instead, precision-based immunotherapeutic strategies will likely require rational combination approaches aimed at overcoming specific barriers, including impaired antigen presentation, myeloid-driven immunosuppression, and TGF-β-mediated immune exclusion. In this context, macrophage-targeting therapies, TGF-β pathway inhibitors, epigenetic modulators, or adoptive cellular therapies could represent logical avenues to enhance immune responsiveness and may provide a more effective framework for immunotherapy in OS.

## 7. Nanotechnology-Enabled Therapeutic Strategies for Osteosarcoma Treatment and Bone Reconstruction

In recent years, nanotechnology has gained increasing attention as a potential driver of therapeutic innovation in OS. This interest is linked to its capacity to address several limitations of conventional treatments, including systemic toxicity, inefficient drug penetration into tumour tissue, and the emergence of chemoresistance. Instead of relying only on dose escalation or new cytotoxic agents, nanotechnology-based approaches introduce a fundamentally different strategy, focused on improving the delivery and distribution of therapy within the body. A key strength of nanotechnology is the ability of nanoscale carriers to preferentially accumulate within tumour tissues and to release therapeutic drugs in a more controlled and localised manner. By exploiting distinctive physicochemical features, such as size, surface charge, and functionalisation, nanoparticles can be engineered to enhance drug stability, prolong circulation time, and reduce off-target exposure. In addition, these platforms offer the possibility of integrating therapeutic and diagnostic functions within a single system, enabling real-time imaging alongside treatment and supporting the growing concept of theragnostic. In the context of OS, where standard chemotherapy remains limited to dose toxicities and suboptimal biodistribution, nanotechnology-based strategies provide an opportunity to redirect precision treatment. By improving tumour selectivity and minimising systemic adverse effects, these approaches have the potential to enhance therapeutic efficacy while preserving patient safety.

Beyond their role as drug delivery systems, advanced bioengineered nanomaterials and hybrid biomaterial platforms are increasingly designed to actively interact with the bone tumour microenvironment while simultaneously supporting post-resection bone regeneration. Recent studies demonstrate that multifunctional nanostructured scaffolds can integrate local antitumour activity with osteoconductive and osteoinductive properties, thereby addressing both tumour eradication and skeletal reconstruction following surgical resection [137,138,139].

### 7.1. Magnetic Nanoparticles and Magnetic Hyperthermia Therapies

Superparamagnetic iron oxide nanoparticles (SPIONs) have attracted considerable interest as multifunctional nanoplatforms for OS treatment, mainly because of their magnetic responsiveness, acceptable biocompatibility, and ease of surface modification. When subjected to an alternating magnetic field, typically using a frequency in the range of 100–500 kHz and a field strength of approximately 10–20 kA/m, SPIONs can generate heat through Néel and Brownian relaxation processes. This localised temperature increase generally falls within the hyperthermic window of 39–45 °C, which is sufficient to affect tumour cells while limiting damage to surrounding healthy tissues [140]. Experimental studies in OS models have shown that magnetothermal treatment maintaining temperatures around 42–43 °C can markedly reduce tumour cell viability. These effects are associated with the activation of caspase-dependent apoptosis, mitochondrial dysfunction, and increased intracellular ROS. Notably, magnetic hyperthermia has been reported to act synergistically with conventional chemotherapeutic agents, including cisplatin and doxorubicin, leading to enhanced cytotoxic effects at lower drug doses than those required for chemotherapy alone [141].

Surface coating plays a crucial role in determining the biological behaviour of SPIONs; functionalisation with polymers or biomaterials such as dextran, polyethylene glycol, chitosan, or silica is commonly employed to improve colloidal stability, reduce magnetic aggregation, and modulate surface charge under physiological conditions. In parallel, SPIONs act as efficient T2/T2* MRI contrast agents because their superparamagnetic core creates local magnetic-susceptibility effects that shorten transverse relaxation times and cause signal loss on T2- and T2*-weighted images. This imaging capability enables the use of SPIONs in theragnostic strategies, combining diagnosis and therapy [142]. Despite these advantages, in vivo studies consistently indicate preferential accumulation of SPIONs in reticuloendothelial organs, particularly the liver and spleen, highlighting the importance of optimising surface chemistry and pharmacokinetics to address long-term clearance and translational safety in OS therapy (Table 3).

### 7.2. Mesoporous Bioactive Glasses (MBGs): Ion Release, Drug Delivery, and Bone Regeneration

Mesoporous bioactive glasses (MBGs) have emerged as particularly attractive materials in the context of OS due to their intrinsic bioactivity and their ability to act simultaneously as therapeutic carriers and ion-releasing platforms [143]. A defining feature of MBGs is their ordered mesoporous structure, with reported pore sizes ranging from 2 to 50 nm, coupled with very high specific surface areas that can approach 1000 m^2^/g [143,144]. These structural characteristics directly translate into a high capacity for drug loading and finely tuneable release behaviour. Importantly, MBGs are not biologically inert carriers. Their therapeutic relevance also derives from the gradual dissolution of the glass matrix and the consequent release of bioactive ions. Calcium and silicon ions released from MBGs are well known to support osteogenic processes, promoting osteoblast differentiation and matrix mineralisation [145,146]. At the same time, the incorporation of additional components within MBG-based systems can confer antitumoural effects. In OS models, composite MBG platforms containing magnetic or metal-based elements have demonstrated direct cytotoxic activity, as exemplified by a Mc–Fe_3_O_4_–MBG system showing an IC_50_ value of 12.19 μg/mL against MG63 OS cells [147]. The mesoporous architecture of MBGs also enables highly efficient drug delivery. Several MBG-based formulations have been reported to achieve doxorubicin loading efficiencies up to 90% [148,149]. Drug release from these platforms is strongly influenced by environmental conditions. In particular, acidic environments promote enhanced drug release, with tuneable profiles observed across a wide pH range from 4.4 to 10, a behaviour that is especially relevant given the altered pH conditions typically associated with OS lesions [148].

Beyond their role in drug delivery, MBGs are well suited for post-surgical applications. MBG-based scaffolds combining sustained ion release with high drug-loading capacity have shown the ability, in preclinical models, to suppress residual tumour cell activity while simultaneously supporting new bone formation and scaffold integration [143]. This combination of localised antitumoural action and osteogenic stimulation addresses a key clinical challenge in OS treatment, where effective control of residual disease must be achieved alongside reconstruction of large bone defects following tumour resection (Table 3).

### 7.3. Biodegradable Metal-Based Scaffolds Employed for Bone Reconstruction

Biodegradable metallic scaffolds have attracted growing interest in OS research because they are able to provide temporary mechanical-loading support while progressively degrading in vivo, thereby reducing the long-term complications associated with permanent metallic implants. For this reason, an ideal bioresorbable scaffold must degrade at a rate that is well matched to the process of new bone formation. If degradation is too fast, mechanical stability is lost; if it is too slow, the material may interfere with bone regeneration. Importantly, this balance strongly depends on the material used [150]. Magnesium-based alloys are generally characterised by relatively rapid corrosion, zinc-based systems exhibit slower degradation behaviour, and iron-based materials degrade very slowly, making precise control of degradation kinetics a central design challenge [151].

Iron combines favourable mechanical properties with limited intrinsic corrosion, which has stimulated the development of strategies aimed at accelerating its degradation. Micro-alloying approaches have been proved to be effective in this regard. For example, Fe–Se alloys with selenium contents in the range of 0.2–1.0 wt% have been reported to inhibit OS cell proliferation while maintaining good compatibility with healthy cells. In vitro studies on human 143B OS cells showed a marked reduction in cell viability (<50%) upon exposure to Fe–Se extracts ranging from 0.6 to 1.0, whereas pre-osteoblastic MC3T3–E1 cells retained viability above 75–100%, indicating a favourable therapeutic window [152]. Mechanistically, the antitumoural effect was associated with Se^4+^ ion release and a significant increase in intracellular ROS, leading to apoptosis and loss of mitochondrial function in OS cells.

Magnesium-based platforms have been evaluated more extensively in OS-oriented experimental settings. Antitumoural effects of Mg-based pellets have been assessed in both 2D and 3D in vitro culture systems, and these findings have been further validated in tumour-bearing mouse models, with mechanistic analyses performed at the molecular and cellular levels. Beyond the effects associated with magnesium corrosion alone, Mg implants can also be engineered into multifunctional systems. An illustrative example is provided by AZ31 Mg alloys that are surface-modified with Fe–OH, as they have been shown to enable combined photothermal and chemo-dynamic therapeutic effects [153].

More complex composite designs have also been explored. A low-temperature 3D-printed poly(lactic-co-glycolic acid) or PLGA/Mg multifunctional scaffold was evaluated in vivo using a rat distal femur model, where near-infrared laser irradiation was employed to eliminate residual tumour cells [154]. In this system, the magnesium component contributed both to the photothermal response and to the promotion of bone regeneration. All together, these metal-based scaffolds are particularly attractive because they integrate structural support with time-dependent degradation and can be further optimised through surface modification or composite engineering to meet the dual clinical demands of OS treatment: effective local tumour control and reconstruction of large bone defects following resection (Table 3).

### 7.4. Injectable Hydrogels for Local Therapy and Bone Regeneration

Injectable hydrogel systems have been explored as localised therapeutic platforms for OS, particularly for use within bone defects following tumour resection. Several formulations are designed to undergo rapid in situ gelation and display self-healing behaviour, with reported stress-relaxation half-times in the range of 30–40 s, allowing them to conform efficiently to irregular surgical cavities. These properties further prolonged retention of therapeutic agents at the target site while limiting systemic dispersion [155]. From a therapeutic point of view, hydrogel-based delivery has demonstrated sustained release profiles lasting approximately 14–21 days, resulting in markedly improved local efficacy compared with free-drug administration. In murine OS models involving incomplete tumour resection, drug-loaded hydrogel implants have been associated with tumour growth inhibition rates of about 85–90%, whereas substantially lower inhibition is observed with single-agent or non-localised treatments. Survival analyses in these models further indicate a benefit for localised hydrogel-based therapies, with median survival times extended by roughly 10–15 days relative to control groups [156].

Hydrogels have also been engineered to support bone regeneration alongside tumour suppression. Micro-computed tomography performed 3–4 weeks after implantation has revealed clear increases in bone mineral density and bone mineral content within hydrogel-treated defects. At the molecular level, expression of osteogenic markers, including ALP, COL1, RUNX2, and BGLAP, has been reported to rise by approximately 3–5-fold, indicating active osteogenic differentiation in the regenerated tissue [156]. In addition, in the literature, it has been well highlighted that hydrogel degradation profiles can be tuned over several weeks, aligning with the temporal requirements of bone healing, while maintaining favourable cytocompatibility, with in vitro cell viability typically exceeding 80–85% [157]. Taken together, these findings support the use of hydrogel-based systems as localised therapeutic matrices capable of integrating effective tumour control with bone regeneration in OS (Table 3).

### 7.5. Limitations and Translational Challenges of Nanotechnology-Based Approaches

Despite the strong interest in nanotechnology-based strategies for OS, their transition from experimental models to clinical practice remains far from straightforward. One of the most recurrent issues concerns nanoparticle biodistribution. In vivo, a substantial fraction of administered nanocarriers is sequestered by organs of the reticuloendothelial system, particularly the liver and spleen. This limits the effective dose reaching tumour tissues and raises legitimate questions regarding long-term accumulation and safety.

Tumour targeting represents an additional critical bottleneck. In most designs, nanoparticle accumulation relies on the enhanced permeability and retention effect; hence, this mechanism is highly variable and often markedly attenuated in patients compared with preclinical models. Consequently, therapeutic benefits observed in experimental settings are not always reproduced clinically. In OS, these limitations may be further exacerbated by the dense extracellular matrix and elevated interstitial pressure, which hinder nanoparticle penetration and result in heterogeneous drug distribution within the tumour mass.

Beyond biological limitations, practical and regulatory challenges also need to be considered. The large-scale production of complex nanomaterials remains technically demanding, particularly with respect to batch-to-batch reproducibility and quality control. In addition, incomplete biodegradation and slow clearance of certain nanoplatforms may lead to tissue accumulation over time, underscoring the importance of thorough and long-term safety evaluation. Taken together, these considerations indicate that, while nanotechnology offers compelling opportunities for OS treatment, significant optimisation and clinically driven validation are still required before widespread clinical implementation can be realistically achieved.

## 8. Conclusions and Future Perspectives

Osteosarcoma remains a biologically complex and clinically challenging malignancy, in which decades of limited therapeutic progress reflect extensive genomic instability, pronounced tumour heterogeneity, and strong microenvironmental influences. While conventional chemotherapy continues to represent the backbone of treatment, the accumulating biological evidence reviewed here indicates that meaningful clinical advances are unlikely to arise from single-target strategies.

Among the approaches discussed, combination therapies currently represent the most promising avenue for near-term clinical impact. In particular, strategies integrating standard chemotherapy with targeted agents or immune-modulating approaches appear more likely to overcome pathway redundancy and adaptive resistance mechanisms than monotherapies. In this context, increasing attention is being directed toward the tumour microenvironment, as targeting macrophage polarisation, osteoclast activity, and stromal–tumour crosstalk emerges as a critical complement to tumour-intrinsic interventions.

At the translational level, nanotechnology-based drug delivery systems stand out as one of the most advanced and clinically realistic innovations, offering the potential to improve drug bioavailability, tumour selectivity, and toxicity profiles. These platforms provide a pragmatic route to enhance the efficacy of existing therapies and may reach clinical implementation earlier than many molecularly targeted strategies. By contrast, immunotherapy has so far shown limited efficacy as a stand-alone treatment in OS, largely due to a strongly immunosuppressive tumour microenvironment. Nevertheless, rational combination regimens aimed at restoring immune competence and counteracting immune exclusion remain an important area for future investigation.

Finally, advances in multi-omics profiling and precision oncology frameworks are expected to refine patient stratification and therapeutic decision-making over the longer term. Although their immediate clinical impact is still limited, these approaches hold promise for identifying biologically defined subgroups and guiding the rational design of combination therapies.

Taken together, these considerations underscore the need to move beyond reductionist models and toward integrated therapeutic strategies that account for OS heterogeneity and tumour–microenvironment interactions. Aligning biological insight with rational therapeutic design will be essential to translating emerging discoveries into tangible benefits for osteosarcoma patients.

## Figures and Tables

**Table 1:** Targeted therapies and signalling pathways in osteosarcoma.

Target/Pathway	Representative Agents	Preclinical Evidence	Clinical Evidence	Main Limitations
IGF-1R	Monoclonal antibodies and small-molecule inhibitors	Reduced OS cell proliferation and tumour growth in preclinical models	Limited clinical benefit in early-phase trials	Pathway redundancy and compensatory signalling via insulin receptor
VEGFR/PDGFR (multikinase)	Regorafenib, pazopanib, and sorafenib	Anti-angiogenic activity and delayed tumour progression in vivo	Improved progression-free survival in phase II trials	Predominantly cytostatic effects; transient responses
PI3K/AKT/mTOR	Everolimus and temsirolimus	Growth inhibition and enhanced chemosensitivity in selected models	Variable and inconsistent activity as monotherapy	Strong pathway redundancy; rapid compensatory signalling; tumour heterogeneity
TGF-β/ALK5	Small-molecule ALK5 inhibitors	Reduced primary tumour growth and metastatic burden; immune modulation	Acceptable safety in early-phase trials	Limited efficacy as single agents; need for rational combinations
DNA damage response	PARP inhibitors	Enhanced cytotoxicity when combined with DNA-damaging agents	No clear benefit as monotherapy	Lack of classical homologous recombination deficiency
Epigenetic regulators	HDAC and DNMT inhibitors	Reduced viability and clonogenicity; resensitisation to chemotherapy in combination settings	Modest clinical activity as single agents	Systemic toxicity; absence of predictive biomarkers

**Table 2:** Immunotherapy and immune-modulating strategies in osteosarcoma.

Strategy	Target/Mechanism	Experimental Evidence	Clinical Evidence	Key Challenges
Immune checkpoint inhibition	PD-1/PD-L1 and CTLA-4	Limited immune activation in OS models	Low objective response rates in clinical trials	Immune-suppressed tumour microenvironment; low neoantigen expression
Macrophage activation	Mifamurtide (L-MTP-PE)	Enhanced macrophage-mediated antitumour activity	Improved overall survival when combined with chemotherapy	Benefit restricted to selected patient subsets
Macrophage reprogramming	CSF-1R inhibition, ATRA, and metabolic modulators	Reduced tumour growth and metastasis in preclinical models	Limited or absent clinical validation in OS	Macrophage plasticity; context-dependent effects
Innate immune checkpoint blockade	CD47–SIRPα axis	Increased macrophage-mediated phagocytosis in OS models	No clinical data currently available in OS	Potential hematologic toxicity; target ubiquity
CAR-T cell therapy	GD2 and B7-H3 (HER2 in selected settings)	Potent cytotoxicity in vitro and xenograft models	Limited and transient responses in early clinical experience	Antigen heterogeneity; poor persistence and trafficking
CAR-NK cell therapy	B7-H3, CD70, and MCAM	Effective tumour suppression in preclinical models	Early developmental stage	Short in vivo lifespan; limited persistence

**Table 3:** Nanotechnology-based and biomaterial platforms for osteosarcoma therapy and bone reconstruction.

Platform	Primary Function	Experimental Models	Key Advantages	Main Limitations
Superparamagnetic iron oxide nanoparticles (SPIONs)	Magnetic hyperthermia and imaging	OS cell lines; murine tumour models	Local heat generation; MRI contrast capability; synergy with chemotherapy	Preferential accumulation in liver and spleen; long-term clearance concerns
Mesoporous bioactive glasses (MBGs)	Ion release and drug delivery	In vitro OS models; scaffold implantation	High drug-loading capacity; pH-responsive release; osteogenic ion delivery	Control of degradation kinetics and ion dosage
Biodegradable metal-based scaffolds (Mg, Fe, and Zn)	Load-bearing support and local antitumour effects	2D/3D cultures; rodent bone defect models	Mechanical strength; time-dependent degradation; suitability for large defects	Precise corrosion-rate control; gas formation (Mg); slow degradation (Fe)
Injectable hydrogels	Local drug depot and bone regeneration	Murine post-resection OS models	In situ gelation; sustained release (≈14–21 days); combined tumour control and repair	Limited penetration depth; formulation-dependent stability

## Data Availability

No new data were created or analyzed in this study. Data sharing is not applicable to this article.
